# Quality in Psychiatric Care in a global perspective

**DOI:** 10.1192/j.eurpsy.2023.1166

**Published:** 2023-07-19

**Authors:** A. Schröder, L.-O. Lundqvist

**Affiliations:** Faculty of Medicine and Health, Örebro University, Örebro, Sweden, University Health Care Research Center, Örebro, Sweden

## Abstract

**Introduction:**

Worldwide efforts to standardize instruments measuring quality in psychiatric care are rare. The international project “Quality in Psychiatric Care” (QPC) is a large research programme aiming at adapting the versions of the QPC instrument for patients and staff to different international settings.

**Objectives:**

The aims were to test the psychometric properties and equivalence of dimensionality of the different language versions of QPC and also to describe and compare the quality of psychiatric out-patient, in-patient, forensic in-patient and psychiatric care across different countries.

**Methods:**

The QPC is a family of instruments based on a definition of quality of psychiatric care from the patients perspective with adapted versions for staff. In this project, we used different language versions in three areas for patient and staff: psychiatric out-patient (QPC-OP/OPS), in-patient (QPC-IP/IPS), and forensic in-patient (QPC-FIP/FIPS).

**Results:**

Patients in out-patient psychiatric care in Brazil rated the quality of care higher than Swedish patients. Comparisons of forensic in-patient care (QPC-FIP/FIPS) patients were more critical of the care they received while staff were generally more positive on the quality of care provided in both Denmark and Sweden. Quality of in-patient care (QPC-IP/IPS) in Spain show staff rating lower quality of care than patients and lowest in the secure environment, which the Swedish staff rated low as well. In Indonesia the patients rated lower quality than staff and lowest in the discharge dimension, followed by the participation dimension. Generally, staff and patients were similar in their perceptions of the low quality of participation. Several studies in Turkey, Indonesia, Spain, Faroe Islands and Norway is ongoing.

**Conclusions:**

The psychometric test and validations of the instrument QPC in different language and country versions will assist countries to compare quality of care, quality improvement and permits benchmarking. Since there are few standardized instruments for measuring quality of care in the psychiatric care, the QPC is expected to make an important contribution to the development in the field.

**Disclosure of Interest:**

None Declared

